# MHD Williamson Nanofluid Flow over a Slender Elastic Sheet of Irregular Thickness in the Presence of Bioconvection

**DOI:** 10.3390/nano11092297

**Published:** 2021-09-03

**Authors:** Fuzhang Wang, Muhammad Imran Asjad, Saif Ur Rehman, Bagh Ali, Sajjad Hussain, Tuan Nguyen Gia, Taseer Muhammad

**Affiliations:** 1 School of Mathematical and Statistics, Xuzhou University of Technology, Xuzhou 221018, China; wangfuzhang1984@163.com; 2 Nanchang Institute of Technology, Nanchang 330044, China; 3 College of Computer Science and Technology, Huaibei Normal University, Huaibei 235000, China; 4 Department of Mathematics, University of Management and Technology, Lahore 54770, Pakistan; imran.asjad@umt.edu.pk (M.I.A.); saifurrehman8684@gmail.com (S.U.R.); 5 Department of Applied Mathematics, School of Science, Northwestern Polytechnical University, 127 West Youyi Road, Xi’an 710072, China; baghalisewag@mail.nwpu.edu.cn; 6 School of Aerospace and Mechanical Engineering, Nanyang Technological University, Singapore 639798, Singapore; sajjadgut@gmail.com; 7 Department of Computing, University of Turku, Agora 4th floor, Vesilinnantie 5, 20500 Turku, Finland; 8 Department of Mathematics, College of Sciences, King Khalid University, Abha 61413, Saudi Arabia; tasgher@kku.edu.sa

**Keywords:** nanofluid, bioconvection, thermal conductivity, slender elastic sheet, thermal radiation

## Abstract

Bioconvection phenomena for MHD Williamson nanofluid flow over an extending sheet of irregular thickness are investigated theoretically, and non-uniform viscosity and thermal conductivity depending on temperature are taken into account. The magnetic field of uniform strength creates a magnetohydrodynamics effect. The basic formulation of the model developed in partial differential equations which are later transmuted into ordinary differential equations by employing similarity variables. To elucidate the influences of controlling parameters on dependent quantities of physical significance, a computational procedure based on the Runge–Kutta method along shooting technique is coded in MATLAB platform. This is a widely used procedure for the solution of such problems because it is efficient with fifth-order accuracy and cost-effectiveness. The enumeration of the results reveals that Williamson fluid parameter λ, variable viscosity parameter $\Lambda_{\mu }$ and wall thickness parameter ς impart reciprocally decreasing effect on fluid velocity whereas these parameters directly enhance the fluid temperature. The fluid temperature is also improved with Brownian motion parameter Nb and thermophoresis parameter Nt. The boosted value of Brownian motion Nb and Lewis number Le reduce the concentration of nanoparticles. The higher inputs of Peclet number Pe and bioconvection Lewis number Lb decline the bioconvection distribution. The velocity of non-Newtonian (Williamson nanofluid) is less than the viscous nanofluid but temperature behaves oppositely.

## 1. Introduction

Nanofluids have a lot of thermophysical attributes like improved heat conductivity, heat diffusivity, and viscosity as opposed to their common base liquids, such as oil or water. Nanofluids applications embrace mass and thermal transportation in engineering and industrial appliances, coolant in automotive electronics, such as microscale, microchips, etc. [1]. The idea of the nanofluid was introduced for the first time by Choi [2] in 1995 for enhance the heat transfer rate. By using the FEM approach, Ali et al. [3] investigated the axisymmetric nanofluid stream on unsteady magnetohydrodynamic through an extending sheet with thermal diffusion. Over a slippery extending surface submerged in a permeable medium, theoretical research for unstable and steady magnetohydrodynamic radiating nanofluid flow research by Farooq et al. [4]. Magnetohydrodynamic mixed convection of non-uniform stream with micropolar nanofluid over a stretching energy emission surface of heat source in presence with thermo-diffusion and multi slip impact investigated by Sohaib et al. [5]. MHD effect on the unstable stream of tangent hyperbolic nanofluid past a operating chemical reaction studied by Gharami et al. [6]. Aslani et al. [7] investigated the magnetohydrodynamic effects of micro-magnetorotation analytical solutions and stability analysis for Poiseuille micropolar flow. Numerical evaluation of the nanofluid flow of Ag-water in thermal efficiency and thermodynamic considerations described by Yang et al. [8]. Gkountas et al. [9] analyzed the effect of the nanoparticles interfacial layer on heat transmission in a printed-circuit heat exchanger using an Al2O3-water nanofluid. The latest survey has been encapsulated of nanofluid and its wide applications can be seen [10,11,12].

In a wide range of technical, automobile, and regular housekeeping machinery, non-Newtonian nanofluids are used. Non-Newtonian fluids are those fluids that do not obey Newton’s viscosity law, i.e., continuous viscosity independent of stress. Under the impact of external forces, the viscosity of non-Newtonian fluids can change to either being more fluid or more solid [13]. Mathematicians across the world are testing new research models to introduce new non-Newtonian nanofluid properties through their endless efforts. Williamson (1929) addressed pseudoplastic mass transfer and recommended a governing equation to explain the flow of pseudoplastic fluid and test the results by laboratory experiments [14]. Analysis of Williamson nanofluid with the inducement of bioconvection microorganism and activation energy comprising unsteady transient slip motion along the boundaries was taken by Aldabesh et al. [15]. Yusuf et al. [16] investigated the results of the slip effects and entropy production of Williamson fluid via a permeable wall with chemical compound across DTM on oriented MHD flow. The investigation was done on the Forchheimer Williamson visco-elastic fluid stream through non-linearly extending sheet and entropy production and implications of dual chemical process on magnetohydrodynamics taken by Rasool et al. [17]. Rana et al. [18] analyzed the microbes swimming in nano-bioconvective Williamson fluid’s blood flow. The researcher utilized the non-Newtonian fluid models (e.g., hyperbolic tangent fluid, Powell Eyring fluid, Casson fluid, Williamson fluid, etc.) were utilized to quantify blood flow in the cardiovascular system because these fluids offer a more detailed thinning component. Srinivasulu et al. [19] studied the impact of the magnetic field on Williamson nanofluid flow, heat, and mass transfer through an extending surface. In this work, they investigate the effect of magnetic field on the Williamson nanofluid through an extending sheet having convective constraints. Shateyi et al. [20] investigated the numerical analysis of magnetohydrodynamics with boundary layer stream of Williamson fluid through a extending surface. In this work, they investigate the unsteady free convection constraints stream of incompressible electrically manage Williamson fluid past an extending surface saturated having a porous medium. Sarada et al. [21] studied the influence of magnetohydrodynamics on the thermal transport behavior of a non-Newtonian fluid flow over a stretching sheet in the presence of local thermal non-equilibrium. Hayat et al. [22] studied through melting thermal transport, the two-dimensional flow of Williamson nanofluid across a non-linear variable thickness surface. Hayat et al. [23] investigated the MHD Williamson nanofluid flow over an exponentially porous stretched surface transfers heat and mass. The rate of heat and mass transfer in MHD Williamson nanofluid flow across an exponentially porous stretched surface susceptible to heat generation or absorption and mass suction is investigated in this research. Hashim et al. [24] analyzed the MHD transient flow of Williamson nanofluids with convective thermal transport has many solutions. The physical properties of a non-Newtonian Williamson fluid flow, as well as heat transmission in the presence of suspended nanoparticles, are investigated in this paper using a two-dimensional numerical simulation.

Bioconvection is the study of structure formation built up due to motion of the swimming microorganism. Usually, the gyrotactic microorganism form a dense layer on the upper part of liquid and create density variation. The heavy layer breaks, and the microorganism come down to create a current of upward and downward motion. This phenomena is called bioconvection. In the modern era of biotechnology along with nanofluids are employed in the diagnosis of various dangerous diseases. Firstly, in 1961 Platt [25] studied the bioconvection structure in the cultures of swimming organisms. Jawad et al. [26] investigated the magnetohydrodynamic bioconvection Darcy–Forchheimer stream of Casson nanofluid past a rotating disk in the presence of entropy optimization. In this work, they analyzed Darcy–Forchheimer’s 3-D bioconvection Casson nanofluid stream deserved by a turning disk having entropy optimization. Zuhra et al. [27] studied the gyrotactic microorganism due to magnetohydrodynamic nanofluid stream by using a convectively heated surface. Khan et al. [28] investigated the bioconvection and numerical simulations on magnetohydrodynamic stream by using an upper paraboloid sheet of revolution. In this model, they analyzed the boundary layer stream aspect of generalized magnetic Newtonian fluid because of paraboloid rotation under bioconvection. Ferdows et al. [29] studied the bioconvection magnetohydrodynamic stream and thermal transport of nanofluid over an extending surface. In this work, they investigate the MHD stream dissipative nanofluid in the presence of gyrotactic microorganisms with an exponentially stretching surface. By approach of Finite element method with microorganism, Ali et al. [30] analyzed the impacts of Stefan blowing on Cattaneo–Christove and thermal radiation aspects for nanofluid stream. Yusuf et al. [31] entropy generation and magneto-bioconvection flow of Williamson nanofluid across an inclined plate with Gyrotactic microorganisms.

Thermal radiation impact in heat transfer has a wide range of applications in thermal engineering, such as gas turbines, nuclear power plants, and numerous propulsion devices for space vehicles, satellites, missiles, and aircraft. Moreover, thermal radiation impact is certain for space applications where few devices are sketched to move levels to attain high thermal efficiency at high-temperature. For this reason, the radiation impact is important while determining thermal impacts in the processes having high-temperature [32]. Pop et al. [33] described the effects of thermal radiation on the stream near the stagnation point of an extending surface. In this work, the boundary surface, and its viscosity enhances having the radiation. Kumar et al. [34] studied the non-uniform hydromagnetic stream of nanofluid through an inclined permeable extending sheet having thermal radiation. In this work, they investigated the non-uniform MHD stream of Eyring–Powell nanofluid impacts of heat radiation and chemical reaction are assumed over an inclined porous extending surface. Shoaib et al. [35] investigated the numerical simulation for rotating stream of magnetohydrodynamic hybrid nanofluid in the presence of thermal radiation past an extending surface. Rehman et al. [36] investigated the numerical simulation effect of buoyancy and thermal radiation on MHD nanofluid stream past a stretching surface. Ghasemi et al. [37] studied the thermal radiation impacts on the magnetohydrodynamic stagnation point flow of a nanofluid past an extending sheet. By using the FEM technique, Khan et al. [38] studied the multi-slip impacts on magnetohydrodynamic viscous nanofluid stream past a porous extending surface in the presence of radiation. Benos et al. [39] studied the thermal transport of a continually stretching and shrinking sheet with mass transpiration of the horizontal boundary is affected by magnetohydrodynamic and radiation processes. The goal of this study is to look at heat transfer for both stretching and shrinking sheets with a horizontal wall that allows for mass transpiration. Ghadikolaei et al. [40] analyzed the effect of non-linear thermal radiation MHD nanofluid stream having joule heating impact with the inclined porous extending surface. Arifuzzaman et al. [41] studied the MHD radiative fourth-grade fluid hydrodynamic stability and heat and mass transfer flow study via porous plate with chemical reaction. The purpose of this study is to investigate the heat and mass transfer characteristics of a naturally convective hydromagnetic flow of fourth-grade radiative fluid produced by a vertical porous plate.

A glance at the existing literature convinces that heat and mass transfer across an irregular geometry is rarely discussed with bioconvection for non-Newtonian Williamson nanofluids. The flow, temperature and concentrations distributions are investigated owing to a slender stretching sheet of varying thickness. Temperature dependent viscosity, thermal conductivity and radiations develop significant aspects of this work. The central idea pertains to the improvement in thermal transport for heat exchangers of compact heat density. The heat transfer is limited to conventional modes with less thermal conductivity of base fluid. Present work is perceived to enhance thermal conductivity of bulk fluid with homogeneous mixture of dilute nano particles. Bioconvection of gyrotactic self motive microorganisms is also useful aspect of this communication to avoid possible settling of nano entities. The following queries are sought:

- How do the transport behaviors change for Newtonian fluid as compared with non-Newtonian (Williamson fluid)?
- What is the impact of nanofluid slip parameters (Brownian and thermophoresis) on temperature?
- How do the bioconvection parameters influence the flow of fluids?

It is revealed that velocity for non-Newtonian (Williamson nanofluid) is solute as compare to Newtonian fluid, whereas the temperature behaves oppositely. Additionally, the biconvection parameter Rb and Nr impede the fluids flow. The nanoparticles slip parameters Nt and Nb enhance the thermal conductivity.

## 2. Statement of the Problem

**Figure 1:**
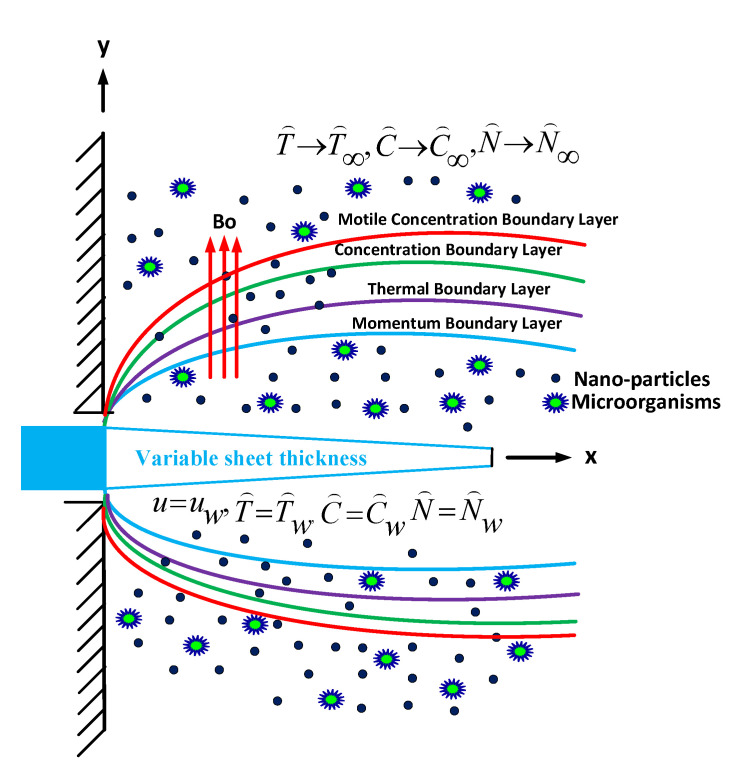
Flow diagram with variable thickness model.

We consider a steady magnetohydrodynamic two-dimensional laminar flow of an incompressible Williamson nanofluid over an extending surface issuing from a slit at the origin through which the sheet is drawn through the fluid medium. Thermal distribution through Williamson nanofluid transportation owing to the non-linear stretch in horizontally, lying slender sheet is taken into consideration. It is considered that the sheet is not flat. Magnetic field $B\left(x\right)=B_{0}{\left(x+a\right)}^{\frac{n-1}{2}}$ along the transverse direction of flow, where $B_{0}$ is uniform magnetic field. The fluid is considered to be marginally conducting a very small Reynolds number, hence the induced magnetic field is neglected. The Williamson nanofluid demonstrating a temperature dependency for thermal conductivity and dynamic viscosity operating through the non-linearly slender surface of variable thickness $\epsilon \left(x\right)=2c{\left(x+a\right)}^{\frac{1-n}{2}}$, that is heated constantly with wall temperature $T_{w}$. The stretched surface is along the positive x−axis with variable velocity of the form $u_{x}\left(x\right)=b{\left(x+a\right)}^{n}$. In this problem *a*, *b* and *c* are constants and used for the utterance of ϵ(x) and $u_{x}\left(x\right)$, to indicate the slender surface dynamical response and its geometry. The exponent *n* delegates the velocity power parameter. In this problem, the sheet is adequately thin in contrast to its width ϵ(x). A mild diffusion of gyrotactic microorganisms is perceived as independent of the nanoparticles. Nanoparticles are mixed in the base fluid homogeneously. As systematically, illustrated in Figure 1. The equation of continuity, momentum, energy, concentration, and bioconvection equation of unsteady Williamson nanofluid boundary layer approximation are as follows (see [19,42]):

Equation of continuity

(1) $$\begin{array}{c} \frac{\partial \hat{u}}{\partial x}+\frac{\partial \hat{v}}{\partial y}=0, \end{array}$$

momentum equation

(2) $$\begin{array}{c} \rho_{f}\left(\hat{u}\frac{\partial \hat{u}}{\partial x}+\hat{v}\frac{\partial \hat{u}}{\partial y}\right)=\sqrt{2}\Gamma \mu_{f}\left(\frac{\partial \hat{u}}{\partial y}\right)\left(\frac{\partial^{2}\hat{u}}{\partial y^{2}}\right)+\mu_{T}\left(\frac{\partial^{2}\hat{u}}{\partial y^{2}}\right)+\left(\frac{\partial \mu_{T}}{\partial \hat{T}}\right)\left(\frac{\partial \hat{T}}{\partial y}\right)\left(\frac{\partial \hat{u}}{\partial y}\right)-\sigma B^{2}\left(x\right)\hat{u}\\ +g[\beta \rho_{f}\left(1-\hat{C_{\infty }}\right)\left(\hat{T}-\hat{T_{\infty }}\right)-\left(\rho_{p}-\rho_{f}\right)\left(\hat{C}-\hat{C_{\infty }}\right)-\left(\rho_{m}-\rho_{f}\right)\left(\hat{N}-\hat{N_{\infty }}\right)], \end{array}$$

energy equation

(3) $$\begin{array}{c} {\left(\rho C_{p}\right)}_{f}\left(\hat{u}\frac{\partial \hat{T}}{\partial x}+\hat{v}\frac{\partial \hat{T}}{\partial y}\right)=k_{T}\left(\frac{\partial^{2}\hat{T}}{\partial y^{2}}\right)+\tau \left[D_{B}\frac{\partial \hat{C}}{\partial y}\frac{\partial \hat{T}}{\partial y}+\frac{D_{T}}{\hat{T_{\infty }}}{\left(\frac{\partial \hat{T}}{\partial y}\right)}^{2}\right]+\left(\frac{\partial k_{T}}{\partial T}\right){\left(\frac{\partial \hat{T}}{\partial y}\right)}^{2}+\frac{\partial q_{r}}{\partial y}, \end{array}$$

concentration equation

(4) $$\begin{array}{c} \hat{u}\frac{\partial \hat{C}}{\partial x}+\hat{v}\frac{\partial \hat{C}}{\partial y}-D_{B}\frac{\partial^{2}\hat{C}}{\partial y^{2}}=\frac{D_{T}}{\hat{T_{\infty }}}\frac{\partial^{2}\hat{T}}{\partial y^{2}}, \end{array}$$

microorganism diffusion equation

(5) $$\begin{array}{c} \hat{u}\frac{\partial \hat{N}}{\partial x}+\hat{v}\frac{\partial \hat{N}}{\partial y}+\frac{dW_{c}}{C_{w}-C_{\infty }}\frac{\partial }{\partial y}\left(\hat{N}\frac{\partial \hat{C}}{\partial y}\right)=D_{N}\left(\frac{\partial^{2}\hat{N}}{\partial y^{2}}\right), \end{array}$$

μ represent the coefficient of viscosity, $C_{p}$ signifies the specific heat at temperature dependency thermal conductivity and constant pressure, $\mu_{T}$ indicates the dynamic viscosity, $\hat{T}$ signifies the nanofluid temperature, $k_{T}$ signifies the thermal conductivity.

By consideration, a linear temperature dependent for thermal physical quantities $\left(\mu_{T},\kappa_{T}\right)$ are

(6) $$\mu_{T}=\left(1-\Lambda_{\mu }\left(\frac{\hat{T}-\hat{T_{\infty }}}{\hat{T_{w}}-\hat{T_{\infty }}}\right)\right),k_{T}=k\left(1+\Lambda_{k}\left(\frac{\hat{T}-\hat{T_{\infty }}}{\hat{T_{w}}-\hat{T_{\infty }}}\right)\right),$$

where, non-dimensional quantities $\Lambda_{\mu }$ and $\Lambda_{k}$ signifies the variable viscosity and thermal conductivity parameters, respectively.

The Rosseland approximation for radiation is [43]

(7) $$q_{r}=-\frac{4\sigma_{e}}{3\beta_{R}}\frac{\partial T^{4}}{\partial y},$$

here $\sigma_{e}$ signifies the Stefan–Boltzmann constant and $\beta_{R}$ represents the mean absorption coefficient.The temperature difference in flow, the Taylor series approximation for $T^{4}$ in terms of $T_{\infty }$ is considered given as

(8) $$\begin{array}{c} {\hat{T}}^{4}\approx 4T_{\infty }^{4}\hat{T}-3T_{\infty }^{4}, \end{array}$$

using the relation Equations (7) and (8), we get

(9) $$\begin{array}{c} \frac{\partial q_{r}}{\partial y}=\frac{16\sigma_{e}T_{\infty }^{3}}{3\beta_{R}}\left(\frac{\partial^{2}\hat{T}}{\partial y^{2}}\right). \end{array}$$

The dimensional boundary conditions are [42]

(10) $$\begin{array}{c} \hat{u}\left(x,y\right)=u_{w}=b{\left(a+x\right)}^{n},\hat{v}\left(x,y\right)=0,\;\hat{T}\left(x,y\right)=\hat{T_{w}},\;\hat{C}\left(x,y\right)=\hat{C_{w}},\hat{N}\left(x,y\right)=\hat{N_{w}},\;at\;y=\frac{\varepsilon (x)}{2}, \end{array}$$

(11) $$\begin{array}{c} \hat{u}\left(x,y\right)\to 0,\;\hat{T}\left(x,y\right)\to \infty ,\;\hat{C}\left(x,y\right)\to \infty ,\hat{N}\left(x,y\right)\to \infty \;as\;y\to \infty .\; \end{array}$$

The boundary value problem consisting of Equations (1)–(11) involve partial differentiation. It is very difficult to solve in this form. Therefore the following similarity transforms [42] are utilized.

(12) $$\begin{array}{c} \hat{u}\left(x,y\right)=b{\left(x+a\right)}^{n}\;{\hat{F}}^{\prime }\left(\xi \right),\;\hat{v}\left(x,y\right)=-{\left(\frac{\left(n+1\right)\nu b{\left(x+a\right)}^{n-1}}{2}\right)}^{1/2}\left(\hat{F}\left(\xi \right)+\left(\frac{n-1}{n+1}\right)\xi {\hat{F}}^{\prime }\left(\xi \right)\right),\\ \xi ={\left(\frac{\left(n+1\right)b{\left(x+a\right)}^{n-1}}{2\nu }\right)}^{1/2}y,\;\psi =\left(\frac{2\nu b{\left(x+a\right)}^{n+1}}{\left(n+1\right)}\right)\hat{F}\left(\xi \right),\;\Theta \left(\xi \right)=\frac{\hat{T}\left(x,y\right)-\hat{T_{\infty }}}{\hat{T_{w}}-\hat{T_{\infty }}},\;\\ \Phi \left(\xi \right)=\frac{\hat{C}\left(x,y\right)-\hat{C_{\infty }}}{\hat{C_{w}}-\hat{C_{\infty }}},\zeta \left(\xi \right)=\frac{\hat{N}\left(x,y\right)-\hat{N_{\infty }}}{\hat{N_{w}}-\hat{N_{\infty }}},\; \end{array}$$

where ψ signifies the stream function.

In view of the above appropriate relations Equation (1) is satisfied and Equations (2)–(5), respectively, become:

(13) $$\begin{array}{c} {\hat{F}}^{\prime \prime \prime }-\frac{2n}{n+1}{\hat{F}}^{\prime 2}+\hat{F}{\hat{F}}^{\prime \prime }+\lambda {\hat{F}}^{\prime \prime }{\hat{F}}^{\prime \prime \prime }-\Lambda_{\mu }{\hat{F}}^{\prime \prime \prime }\Theta -\Lambda_{\mu }{\hat{F}}^{\prime \prime }\Theta^{\prime }-\frac{2M}{n+1}{\hat{F}}^{\prime }+\omega \left[\Theta -Nr\Phi -Rb\zeta \right]=0 \end{array}$$

(14) $$\begin{array}{c} \left(1+Rd\right)\Theta^{\prime \prime }+Pr\hat{F}\Theta^{\prime }+\Lambda_{k}\Theta \Theta^{\prime \prime }+\Lambda_{k}\Theta^{\prime 2}+Nb\Theta^{\prime }\Phi^{\prime }+Nt\Theta^{\prime 2}=0, \end{array}$$

(15) $$\begin{array}{c} \Phi^{\prime \prime }+LePr\hat{F}\Phi^{\prime }+\frac{Nt}{Nb}\Theta^{\prime \prime }=0, \end{array}$$

(16) $$\begin{array}{c} \zeta^{\prime \prime }+PrLbF\zeta^{\prime }-Pe\left[\zeta^{\prime }\Phi^{\prime }+\Omega \Phi^{\prime \prime }+\Phi^{\prime \prime }\zeta \right]=0, \end{array}$$

with boundary constraints,

(17) $$\begin{array}{c} \hat{F}\left(\xi \right)=\frac{1-n}{1+n}\varsigma ,\;{\hat{F}}^{\prime }\left(\xi \right)=1,\;\Theta \left(\xi \right)=1,\;\Phi \left(\xi \right)=1,\zeta \left(\xi \right)=1,\;at\;\xi =\varsigma ,\\ {\hat{F}}^{\prime }\left(\xi \right)\to 0,\Theta \left(\xi \right)\to 0,\Phi \left(\xi \right)\to 0,\zeta \left(\xi \right)\to 0,\;\;as\;\xi \to \infty .\; \end{array}$$

where $Nr=\frac{\left(\rho_{p}-\rho_{f}\right)\left(C_{w}-C_{\infty }\right)}{\beta \rho \left(1-C_{\infty }\right)\left(T_{w}-T_{\infty }\right)}$ signifies the buoyancy ratio parameter, $Rb=\frac{\left(\rho_{m}-\rho_{f}\right)\left(N_{w}-N_{\infty }\right)}{\beta \rho \left(1-C_{\infty }\right)\left(T_{w}-T_{\infty }\right)}$ indicate the bioconvection Rayleigh number, $M=\frac{\sigma B_{0}^{2}}{b\rho }$ delegate the magnetic parameter, $\omega =\frac{2g\beta \left(1-C_{\infty }\right)\left(T_{w}-T_{\infty }\right)}{u_{w}^{2}\left(n+1\right)}$ is the mixed convection parameter, $Rd=\frac{16\sigma_{e}T_{\infty }^{3}}{3\beta_{R}k}$ denotes the radiation parameter, $\lambda =\Gamma \sqrt{\frac{{\left(x+a\right)}^{3n-1}b^{3}\left(n+1\right)}{\nu }}$ represents the Williamson parameter, $Le=\frac{k}{D_{B}}$ denotes the Lewis number, $Nb=\frac{\tau D_{B}\left(C_{w}-C_{\infty }\right)}{k}$ signifies the Brownian parameter, $Nt=\frac{\tau D_{T}\left(T_{w}-T_{\infty }\right)}{T_{\infty }k}$ respresents the thermophoresis parameter, $Pr=\frac{(\rho_{Cp})\nu }{k}$ signifies the Prandtl number, $\varsigma =c{\left(\frac{bn+b}{2\nu }\right)}^{\frac{1}{2}}$ is the wall thickness parameter, $Pe=\frac{dW_{c}}{D_{N}}$ delegate the bioconvection Peclet number, $Lb=\frac{k}{D_{N}}$ indicates the bioconvection Lewis number, $\Omega =\frac{N_{\infty }}{\left(N_{w}-N_{\infty }\right)}$ is the density ratio of motile microorganism.

In order to yield simplification, we suppose,

(18) $$\left\{\begin{array}{l} \xi =\eta +\varsigma ,\\ \hat{F}\left(\xi \right)=\hat{F}\left(\eta +\varsigma \right)=f\left(\eta \right),\\ \Theta (\xi )=\Theta (\eta +\varsigma )=\theta (\eta ),\\ \Phi (\xi )=\Phi (\eta +\varsigma )=\phi (\eta ),\\ \zeta (\xi )=\chi (\eta +\varsigma )=\chi (\eta ), \end{array}\right.$$

where f(η) signifies for the dimensionless stream function. $f^{\prime }(\eta )$ represent for dimensionless velocity profile. θ(η) is the dimensionless nanofluid temperature. ϕ(η) signifies for dimensionless nanofluid concentration. χ(η) delegates for the dimensionless density of motile microorganism, and η indicates for the similarity variable.

These transformations modify the Equations (13)–(16) as below

(19) $$\begin{array}{r} f^{\prime \prime \prime }-\frac{2n}{n+1}f^{\prime 2}+ff^{\prime \prime }+\lambda f^{\prime \prime }f^{\prime \prime \prime }-\Lambda_{\mu }f^{\prime \prime \prime }\theta +\Lambda_{\mu }f^{\prime \prime }\theta^{\prime }-\frac{2M}{n+1}f^{\prime }+\omega \left[\theta -Nr\phi -Rb\chi \right]=0 \end{array}$$

(20) $$\begin{array}{r} \left(1+Rd\right)\theta^{\prime \prime }+Prf\theta^{\prime }+\Lambda_{k}\theta \theta^{\prime \prime }+\Lambda_{k}\theta^{\prime 2}+Nb\theta^{\prime }\phi^{\prime }+Nt\theta^{\prime 2}=0, \end{array}$$

(21) $$\begin{array}{r} \phi^{\prime \prime }+LePrf\phi^{\prime }+\frac{Nt}{Nb}\theta^{\prime \prime }=0, \end{array}$$

(22) $$\begin{array}{r} \chi^{\prime \prime }+PrLbf\chi^{\prime }-Pe\left[\chi^{\prime }\phi^{\prime }+\Omega \phi^{\prime \prime }+\phi^{\prime \prime }\chi \right]=0, \end{array}$$

with modified boundary conditions

(23) $$\begin{array}{c} f\left(\eta \right)=\frac{1-n}{1+n}\;\varsigma ,f^{\prime }\left(\eta \right)=1,\theta \left(\eta \right)=1,\;\phi \left(\eta \right)=1,\chi \left(\eta \right)=1,\;at\;\eta =0,\\ f^{\prime }\left(\eta \right)\to 0,\;\theta \left(\eta \right)\to 0,\;\phi \left(\eta \right)\to 0,\;\chi \left(\eta \right)\to 0,\;as\;\;\eta \to \infty .\; \end{array}$$

## 3. Physical Quantities

This segment describes the attributes of important physical quantities of engineering interest. The local skin friction $C_{fx}$, motile density number $Nn_{x}$, Sherwood number $Sh_{x}$, and Nusselt number $Nu_{x}$ as given below

(24) $$\begin{array}{c} C_{fx}=\frac{2\tau_{xy(x,y=\frac{\epsilon (x)}{2})}}{\rho_{f}u_{w}^{2}\left(x\right)},\;Nu_{x}=\frac{\left(x+a\right)q_{T}}{k({\hat{T}}_{w}-T_{\infty })},\;Sh_{x}=\frac{\left(x+a\right)q_{m}}{D_{B}\left(\hat{C}-C_{\infty }\right)},\;Nn_{x}=\frac{\left(x+a\right)q_{n}}{D_{B}\left(\hat{N}-N_{\infty }\right)},\; \end{array}$$

where *k* signifies the thermal conductivity of the nanofluid, and $q_{w}$,$q_{m}$, and $q_{n}$ are the heat flux, mass flux, and motile microorganisms flux, respectively, given by

(25) $$\begin{array}{c} q_{T}=-{\left(k_{T}+\frac{4\sigma_{e}T_{\infty }^{3}}{\beta_{R}}\left(\frac{\partial \hat{T}}{\partial y}\right)\right)}_{y=\frac{\epsilon (x)}{2}},q_{m}=-D_{B}{\left(\frac{\partial \hat{C}}{\partial y}\right)}_{y=\frac{\epsilon (x)}{2}},q_{n}=-D_{N}{\left(\frac{\partial \hat{N}}{\partial y}\right)}_{y=\frac{\epsilon (x)}{2}}. \end{array}$$

Skin friction coefficient in non-dimensional form is

(26) $$\begin{array}{c} C_{fr}=\sqrt{\frac{\left(n+1\right)}{2}}\left(f^{\prime \prime }\left(0\right)+\frac{\lambda }{2}f^{\prime \prime 2}\left(0\right)\right). \end{array}$$

By using Equations (10), (12) and (18), we get the following expressions

(27) $$\begin{array}{c} Nu_{r}=-\sqrt{\frac{n+1}{2}}\left(1+Rd\right)\theta^{\prime }\left(0\right),\;Sh_{r}=-\sqrt{\frac{n+1}{2}}\phi^{\prime }\left(0\right),\;Nn_{r}=-\sqrt{\frac{n+1}{2}}\chi^{\prime }\left(0\right),\; \end{array}$$

where $C_{fr}$, $Nu_{r}$, $Sh_{r}$ and $Nn_{r}$ represent the reduced forms of local skin friction coefficient $C_{fx}$, Nusselt number $Nu_{x}$, Sherwood number $Sh_{x}$, and density number of motile microorganisms $Nn_{x}$, given as

(28) $$\begin{array}{c} C_{fr}=-Re_{x}^{\frac{1}{2}}C_{fx}, \end{array}$$

(29) $$\begin{array}{c} Nu_{r}=Re_{x}^{\frac{-1}{2}}Nu_{x}, \end{array}$$

(30) $$\begin{array}{c} Sh_{r}=Re_{x}^{\frac{-1}{2}}Sh_{x}, \end{array}$$

(31) $$\begin{array}{c} Nn_{r}=Re_{x}^{\frac{-1}{2}}Nn_{x}, \end{array}$$

where $Re_{x}=\frac{\left(x+a\right)u_{w}\left(x\right)}{\nu }$ signifies the local Reynolds number.

## 4. Solution Procedure

The non-linearity characteristics of the fluid model described finally as boundary value formulation in Equations (19)–(22) with initial and boundary constraints (23) can not be solved analytically. In order to seek physical insight of the problem boundary value problem is required to yield a viable solution. The characteristics for heat and mass transportation as influence by the leading parameters help to understand the very nature of the problem. A reliable numerical procedure is sought for this purpose. Several investigators [44,45] employed shooting technique with the Runge–Kutta method. We also harnessed this scheme to achieve the results of the current work. Here, the step size is taken to be h=0.01 and the order of accuracy is $O(h^{5})$. The higher order derivatives involved in the finally governing equations are reduced to construct first order differential system as below:

$\begin{array}{l} z_{1}^{\prime }=z_{2}, \end{array}$

$\begin{array}{l} z_{2}^{\prime }=z_{3}, \end{array}$

$\begin{array}{l} z_{3}^{\prime }=\frac{\left(-1\right)}{\left(1+\lambda z_{3}-\Lambda_{\mu }z_{4}\right)}\left[\frac{-2n}{n+1}z_{2}^{2}+z_{1}z_{3}-\Lambda_{\mu }z_{3}z_{5}-\frac{2M}{n+1}z_{2}+\omega \left(z_{4}-Nrz_{6}-Rbz_{8}\right)\right], \end{array}$

$\begin{array}{l} z_{4}^{\prime }=z_{5}, \end{array}$

$\begin{array}{l} z_{5}^{\prime }=\frac{\left(-1\right)}{\left(1+Rd+\Lambda_{k}z_{4}\right)}\left[Prz_{1}z_{5}+\Lambda_{k}z_{5}^{2}+Nbz_{5}z_{7}+Ntz_{5}^{2}\right], \end{array}$

$\begin{array}{l} z_{6}^{\prime }=z_{7}, \end{array}$

$\begin{array}{l} z_{7}^{\prime }=\left(-1\right)\left[LePrz_{1}z_{7}+\frac{Nt}{Nb}z_{5}^{\prime }\right], \end{array}$

$\begin{array}{l} z_{8}^{\prime }=z_{9} \end{array}$

$\begin{array}{l} z_{9}^{\prime }=\left(-1\right)\left[PrLbz_{1}z_{9}+Pe\left(\Omega z_{7}^{\prime }+z_{7}^{\prime }z_{8}+z_{7}z_{9}\right)\right], \end{array}$

along with the boundary conditions

$\begin{array}{l} z_{1}=\frac{1-n}{1+n}\varsigma ,\;z_{2}=1,\;z_{4}=1,\;z_{7}=1,\;z_{9}=1,\;at\;\eta =0,\;\\ z_{2}\to 0,\;z_{4}\to 0,\;z_{7}\to 0,\;z_{9}\to 0,\;as\;\eta \to \infty .\; \end{array}$

## 5. Results and Discussion

Exploration for bioconvection of Williamson’s nanofluid over a horizontal extending surface with variable thickness is enumerated in presence of magnetic field and thermal radiation. The numerical findings as achieved from the above code are exhibited and elaborated in this segment. The data enlisted in Table 1 and Table 2 for $Re_{x}^{\frac{1}{2}}C_{fx}$ (skin friction) and $Re_{x}^{\frac{1}{2}}Nu_{x}$ (Nusselt number) helped to built confidence in MATLAB script coding for the numerical procedure. Furthermore, Table 3 and Table 4 are constructed to describe the role of prominent variable on $Re_{x}^{\frac{1}{2}}C_{fx}$ and $Re_{x}^{\frac{1}{2}}Nu_{x}$. Information testify that $Re_{x}^{\frac{1}{2}}C_{fx}$ is intensified with *M*, *n*, $\Lambda_{\mu }$, Nr, Rb. Actually, higher inputs of these numbers oppose the flow in the boundary layer regime. Thus, there is a notable differentiation quotient to signify the larger magnitude of skin friction. Additionally, skin friction is diminished against λ, ω. These parameters enhance the velocity of fluid due to buoyancy effects. Nusselt number is enhanced in direct relation to Pr, Rd, $\Lambda_{k}$, and it is decremented when Nb, Nt increased. From Table 5, Sherwood number $Re_{x}^{\frac{1}{2}}Sh_{x}$ seems to be developing with Le and Nb, whereas it recedes against Nt. The incremented role of Pe and Lb on motile density number $Re_{x}^{\frac{1}{2}}Nn_{x}$ is depicted in Table 6, whereas output of Ω for $Re_{x}^{\frac{-1}{2}}Nn_{x}$ is not significant. The graphical results for Williamson’s nanofluid velocity, temperature, concentration of nanoparticles, and microorganism are computed when λ=0.3 (non-Newtonian) and for the case of fluid λ=0 (Newtonian) [46].

**Figure 2:**
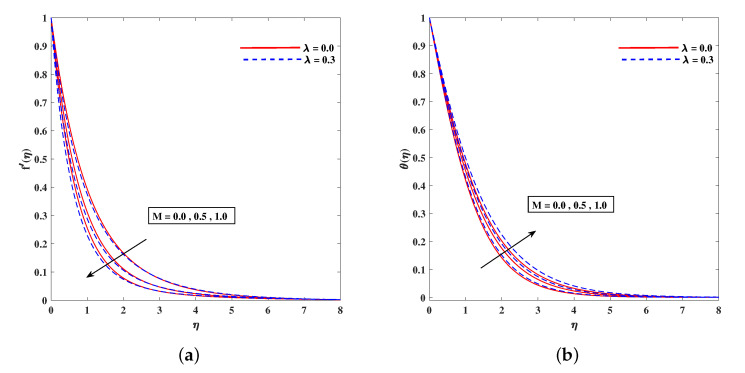
Fluctuation of *M* on velocity $f^{\prime }\left(\eta \right)$ (**a**) and temperature profile θ(η) (**b**).

**Figure 3:**
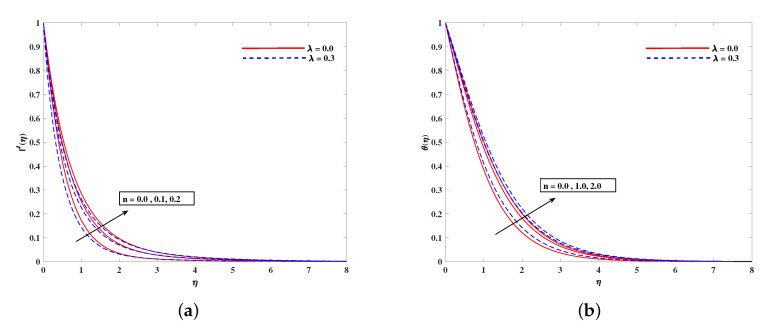
Fluctuation of *n* on velocity $f^{\prime }\left(\eta \right)$ (**a**) and temperature profile θ(η) (**b**).

**Figure 4:**
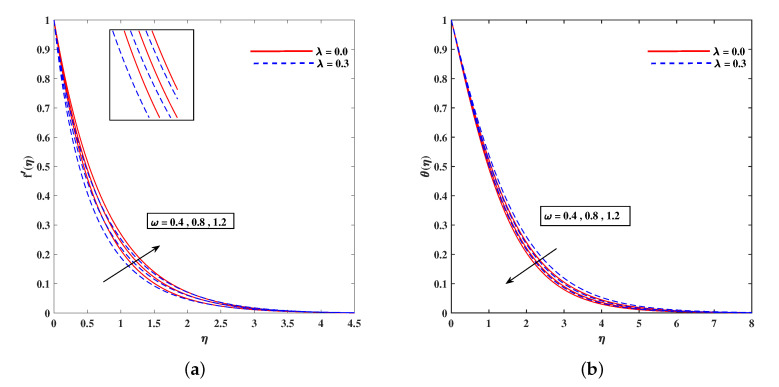
Fluctuation of ω on velocity $f^{\prime }\left(\eta \right)$ (**a**) and temperature profile θ(η) (**b**).

**Figure 5:**
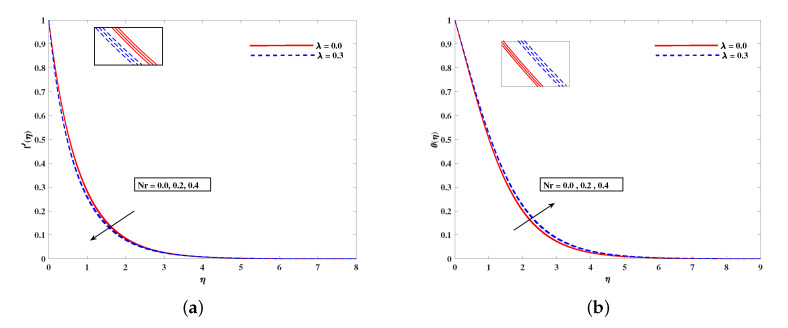
Fluctuation of Nr on velocity $f^{\prime }\left(\eta \right)$ (**a**) and temperature profile θ(η) (**b**).

**Figure 6:**
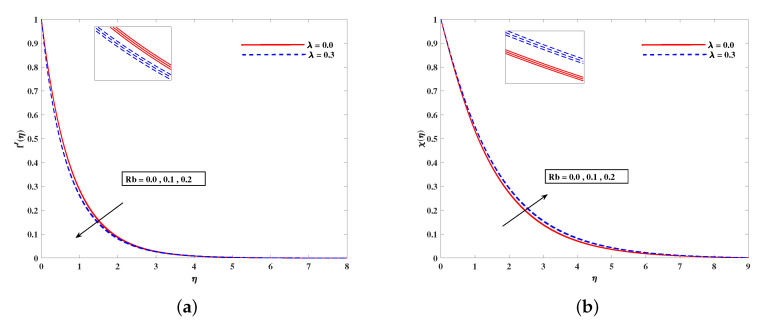
Fluctuation of Rb on velocity $f^{\prime }\left(\eta \right)$ (**a**) and bioconvection profile χ(η) (**b**).

**Figure 7:**
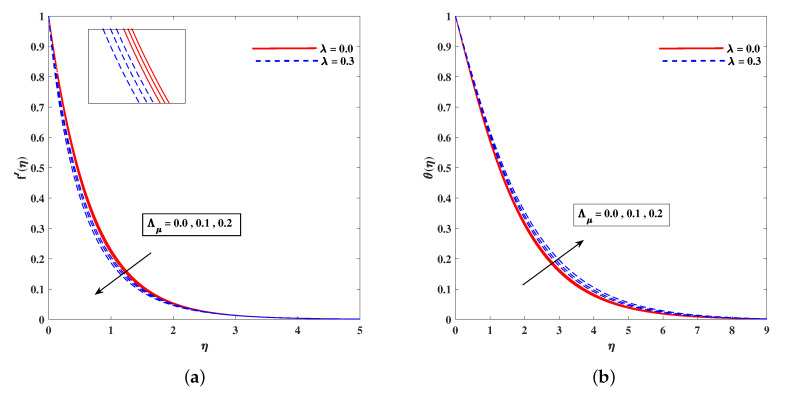
Fluctuation of $\Lambda_{\mu }$ on velocity $f^{\prime }\left(\eta \right)$ (**a**) and temperature profile θ(η) (**b**).

**Figure 8:**
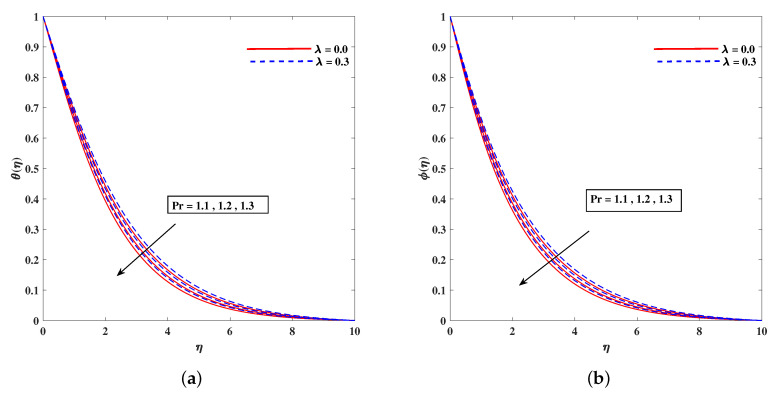
Fluctuation of Pr on temperature θ(η) (**a**) and concentration profile ϕ(η) (**b**).

**Figure 9:**
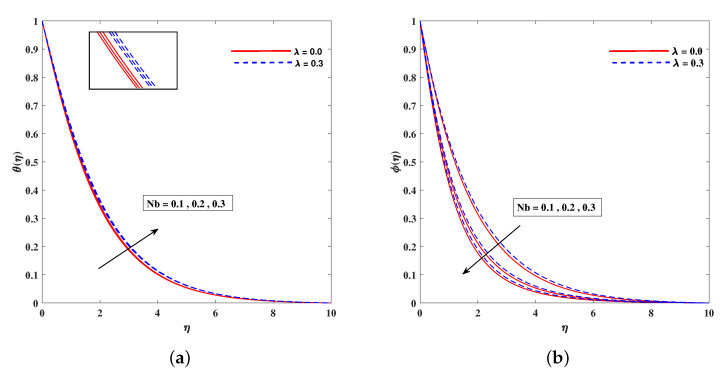
Fluctuation of Nb on temperature θ(η) (**a**) and concentration profile phi(η) (**b**).

**Figure 10:**
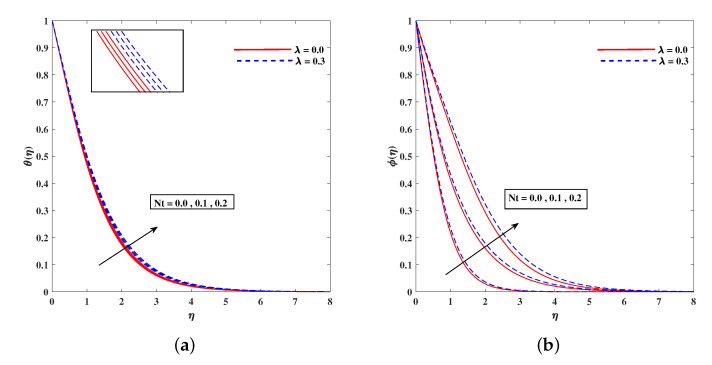
Fluctuation of Nt on temperature (**a**) θ(η) and concentration profile (**b**) ϕ(η).

In Figure 2a, decelerated flow in the face of the growing strength of *M* is caused due to the resistive force (Lorentz). This reactive force comes to play its role when magnetic field interacts with electric field. This slowing of flow helps the conservation for loss of kinetic energy to heat energy and, hence, the enhancement in temperature θ(η) is resulted in Figure 2b. The growing inputs of *n* (non-linear index) provide boosting to $f^{\prime }\left(\eta \right)$ (velocity) and θ(η) (temperature) as depicted, respectively, in Figure 3a,b. From Figure 4a,b, it is seen that intensified mixed convection parameter ω enhances $f^{\prime }\left(\eta \right)$ and it recedes θ(η). The larger values of ω correspond to stronger buoyancy effects to enhance the velocity. The buoyancy ratio parameter Nr depreciated the flow $f^{\prime }$ but it boosted θ as to be perceived from Figure 5a,b. Figure 6a,b portrait the role of bioconvection Rayleigh number Rb on $f^{\prime }$ and θ. The strength of Rb is responsible to decrease $f^{\prime }\left(\eta \right)$ but it promotes θ(η). These two parameters are reciprocal to $\left(T_{w}-T_{\infty }\right)$. The buoyancy effects are reduced to show the flow and raise the temperature in the boundary layer regime. Similar to Rb the impact of the parameter $\Lambda_{\mu }$ is observed on $f^{\prime }\left(\eta \right)$ and θ(η) in Figure 7a,b. Figure 8a,b demonstrate decline in temperature and concentration profile when Prandtl number is enhanced. Actually, Prandtl number is inversely related to thermal diffusivity and, hence, it reduces the two quantities. As noticed from Figure 9a,b, the intensified Brownian motion parameter Nb imparts directly proportional and influence on θ(η), and it recedes the concentration reciprocally. The apart and random transport of nano entities contribute to heat energy and that festally dispersed particles resulted in the lower concentration. Thermophoretic effect signifies the transport of nano particles from higher degree to lower one, as a consequence that temperature θ(η) and concentration ϕ(η) are boosted up in direct relation with Nt(Thermophoretic parameter) as indicated in Figure 10a,b.

**Figure 11:**
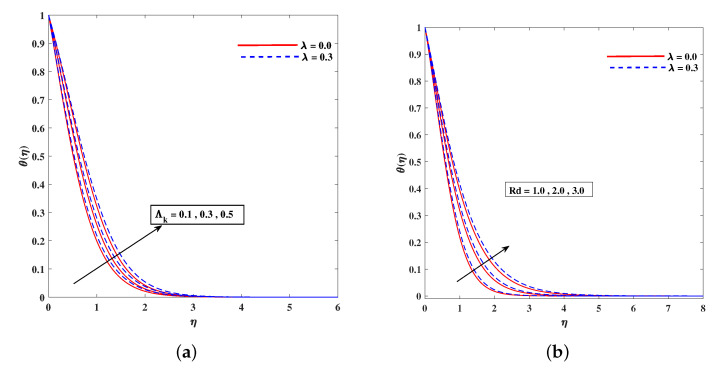
Fluctuation of (**a**) $\Lambda_{k}$ and (**b**) Rd on temperature profile θ(η).

**Figure 12:**
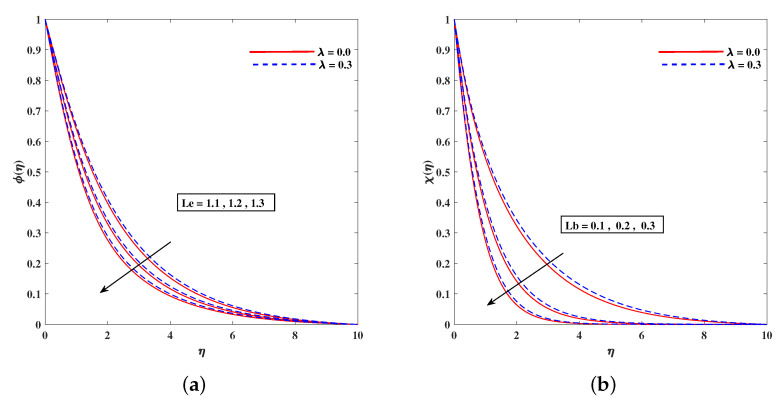
Fluctuation of (**a**) Le on concentration ϕ(η) and (**b**) Lb on bioconvection profile χ(η).

**Figure 13:**
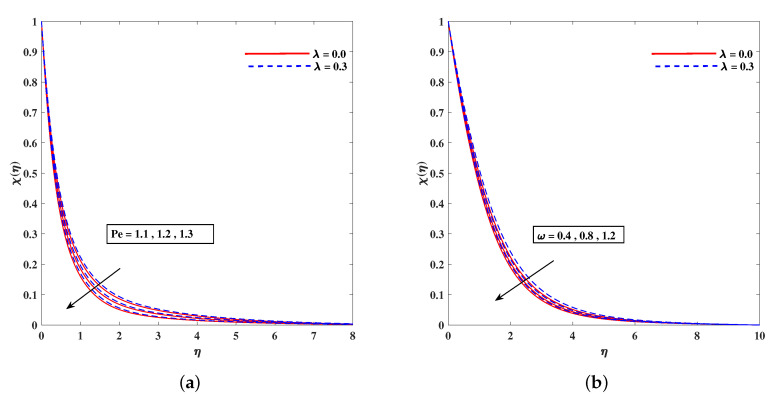
Fluctuation of Pe (**a**) and ω (**b**) on bioconvection profile χ(η).

**Figure 14:**
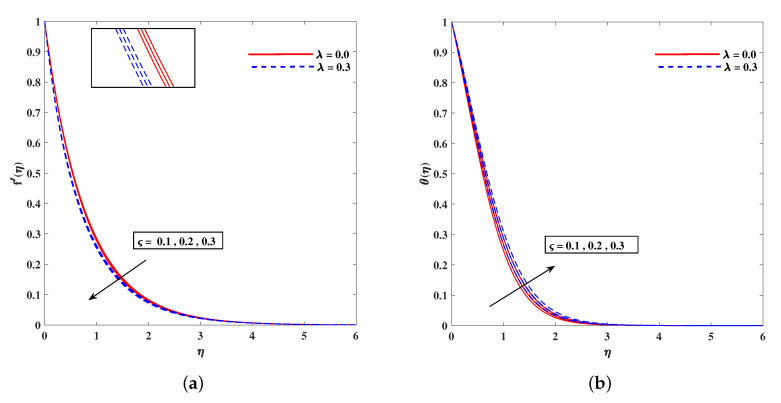
Fluctuation of ς on velocity (**a**) and temperature profile θ(η) (**b**).

Figure 11a,b demonstrates the rising behavior of fluid temperature due to variable thermal conductivity parameter $\Lambda_{k}$ and radiation parameter Rd. Figure 12a sketches that role of enhancing Lewis number Le to reduce nanoparticle concentration ϕ(η) diffusivity coefficient $Le=\frac{k}{D_{B}}$. It is to mention that theoretically, the nanoparticles are taken for metals or metal oxides of size less than 100 nm. A dilute concentration of nanoparticles is taken. Similarly, Figure 12b demonstrates the decline in microorganism distribution χ(η) in retrospective to Lb (bioconvection Lewis number). The intensifying inputs of Peclet number Pe and difference parameter ω play a declying impacts on χ(η) as plotted in Figure 13a,b. Figure 14a portrait the influence of extending wall thickness parameter ς on velocity $f^{\prime }\left(\eta \right)$. It is observed that the larger thickness parameter the velocity curve lowers. The temperature of the fluid rises up when wall thickness parameter is incremented. The graphical pattern of θ(η) in response to ς is shown in Figure 14b.

## 6. Conclusions

The implications of temperature-dependent viscosity and thermal conductivity for heat and mass transport of Williamson nanofluids across a non-linear stretching sheet of irregular thickness are examined when bioconvection of microorganisms is incorporated. Because of the inherent non-linear characteristics of the formulation developed for the problem, a numerical code based on the Runge–Kutta method is run on the Matlab platform. A reasonable agreement is achieved among the past and current studies to validate the results. The notable outcomes are presented below:

- The flow velocity enhances with higher inputs of the power index parameter *n* and mixed convection parameter ω and it declines against the increments in ς, *M*, Nr, Rb, and $\Lambda_{\mu }$, because these parameters are responsible for decelerating the flow;
- The temperature field rises directly with ς, *M*, *n*, Nr, $\Lambda_{\mu }$, Nb, Nt, $\Lambda_{k}$, and Rd and it diminished against rising values of ω and Pr, and the concentration is boost when Nt is increased and it recedes against the incremented values of Nb and Le;
- The microorganism density is depreciated when the parameters Lb, Pe, and ω are given higher inputs;
- The skin friction is intensified with *M*, *n*, $\Lambda_{\mu }$, Nr, Rb, and it is diminished against λ, ω. However, the skin friction is diminished against λ, and ω due to the accelerated flow;
- Nusselt number is enhanced in direct relation to Pr, Rd, $\Lambda_{k}$. As these parameters reduce the temperature distribution, and it is decremented when Nb, Nt increased because the higher inputs of the parameters Nb and Nt enhanced temperature distribution to reduced Nusselt number.

## Figures and Tables

**Table 1:** Comparison of $C_{fx}Re_{x}^{\frac{1}{2}}$ (skin friction coefficient) for different values of *n* and variable thickness ς, when ignored other parameters.

n	Fang [47]		Wakif [42]		(Our Results)	
	ς=0.25	ς=0.5	ς=0.25	ς=0.5	ς=0.25	ς=0.5
10	1.0603	1.1433	1.060324666	1.143320620	1.060343	1.143333
9.0	1.0589	1.1404	1.058915794	1.140392519	1.058934	1.140405
7.0	1.0550	1.1323	1.055044823	1.132285178	1.055062	1.132298
5.0	1.0486	1.1186	1.048611306	1.118590381	1.048628	1.118602
3	1.0359	1.0905	1.035868282	1.090492254	1.035883	1.090503
2.0	1.0234	1.0614	1.023407744	1.061402505	1.023420	1.061412
1.0	1.0000	1.0000	1.000000000	1.000000000	1.000000	1.000000
$\frac{1}{2}$	0.9799	0.9338	0.979944970	0.933825410	0.979949	0.933831
0.0	0.9576	0.7843	0.957643151	0.784279231	0.957644	0.784282
$-\frac{1}{3}$	1.0000	0.5000	1.000000000	0.500000000	1.000000	0.500000

**Table 2:** Comparison of the Nusselt number $Nu_{x}Re_{x}^{-1/2}$, when n=1, for different values of Pr and all others parameter are zero.

Pr	Wang [48]	Mabood [49]	Wakif [42]	(Our Results)
0.7	0.4539	0.4539	0.453916157	0.454447
2.0	0.9114	0.9114	0.911357683	0.911353
7.0	1.8954	1.8954	1.895403258	1.895400
20	3.3539	3.3539	3.353904143	3.353902
70	6.4622	6.4622	6.462199531	6.462198

**Table 3:** Effect of various physical parameters over skin friction coefficient $Re_{x}^{\frac{1}{2}}C_{fx}=-\sqrt{\frac{n+1}{2}}\left(f^{\prime \prime }\left(0\right)+\frac{\lambda }{2}f^{\prime \prime }{\left(0\right)}^{2}\right)$.

*M*	*n*	λ	ω	$\Lambda_{\mu }$	Nr	Rb	$C_{\mathrm{fr}}$
0.5	1	0.3	0.4	0.1	0.3	0.1	1.0581
1.0							1.2521
1.5							1.4208
	1.0						1.0581
	2.0						1.1422
	3.0						1.2498
		0.3					1.0581
		0.4					1.0370
		0.5					0.9900
			0.4				1.0581
			0.5				1.0203
			0.6				0.9832
				0.1			1.0581
				0.2			1.1556
				0.3			1.5167
					0.3		1.0581
					0.4		1.0815
					0.5		1.1053
						0.1	1.0581
						0.2	1.0751
						0.3	1.0923

**Table 4:** Effect of various physical parameters over Nusselt number $Re_{x}^{\frac{-1}{2}}Nu_{x}=-\sqrt{\frac{n+1}{2}}\left(1+Rd\right)\theta^{\prime }\left(0\right)$.

Pr	Rd	$\Lambda_{k}$	Nb	Nt	${\mathrm{Nu}}_{r}$
1.1	1	0.1	0.1	0.1	0.7872
1.2					0.8302
1.3					0.8718
	1.0				0.7872
	2.0				0.9761
	3.0				1.1388
		0.1			0.7872
		0.2			0.7909
		0.3			0.7948
			0.1		0.7872
			0.2		0.7672
			0.3		0.7435
				0.1	0.7872
				0.2	0.7622
				0.3	0.7363

**Table 5:** Effect of various physical parameters over Sherwood number $Re_{x}^{\frac{-1}{2}}Sh_{x}=-\sqrt{\frac{n+1}{2}}\phi^{\prime }\left(0\right)$.

Le	Nt	Nb	${\mathrm{Sh}}_{r}$
0.3	0.1	0.1	0.1876
0.4			0.2361
0.5			0.2849
	0.1		0.1876
	0.2		0.1516
	0.3		0.1173
		0.5	0.1876
		0.6	0.1951
		0.7	0.2006

**Table 6:** Effect of various physical parameters over density of motile microorganism $Re_{x}^{\frac{-1}{2}}Nn_{x}=-\sqrt{\frac{n+1}{2}}\chi^{\prime }\left(0\right)$.

Pe	Lb	Ω	${\mathrm{Nn}}_{r}$
0.1	0.3	0.1	0.2403
0.2			0.2564
0.3			0.2728
	0.3		0.2403
	0.4		0.2874
	0.5		0.3343
		0.1	0.2403
		0.3	0.2423
		0.5	0.2444

## Data Availability

All the data contained within the manuscript.

## Abbreviations

The following abbreviations are used in this manuscript:

$\hat{u}$	Fluid velocity components along x−axis
$\hat{N}$	Density of motile microorganism
$\hat{v}$	Fluid velocity components along y−axis
${\hat{C}}_{w}$	Concentration at surface
*g*	Gravitational acceleration
$D_{B}$	Brownian diffusion coefficient
$\rho_{f}$	Fluid density
$D_{N}$	Diffusivity of microorganisms
$\rho_{p}$	Mass density of nanoparticles
$D_{T}$	Thermophoretic diffusion coefficient
$C_{fx}$	Skin friction
$\hat{T_{w}}$	Temperature at surface
$Nu_{x}$	Nusselt number
$Sh_{x}$	Sherwood number
μ	Dynamic viscosity
ν	Kinematic viscosity
Nt	thermophoresis parameter
$\hat{N_{w}}$	Density of motile microorganism at surface
${\hat{D}}_{T}$	Thermophoretic diffusion coefficient
$\hat{T_{\infty }}$	Ambient temperature
Cp	Specific heat
$B_{0}$	Uniform magnetic field
λ	Fluid parameter
$\hat{C_{\infty }}$	Ambient concentration
$k_{T}$	Thermal conductivity
$\hat{N_{\infty }}$	Ambient motile microorganism
$\hat{C}$	Nanoparticles concentration
$W_{c}$	Constant maximum cell swimming speed
τ	The ratio of heat capacities of nanoparticles
Pr	Prandtl number
